# Immunitarianism: defence and sacrifice in the politics of Covid-19

**DOI:** 10.1007/s40656-021-00384-9

**Published:** 2021-02-22

**Authors:** Btihaj Ajana

**Affiliations:** Department of Digital Humanities, King’s College London, London, UK

**Keywords:** Covid-19, Coronavirus, Immunity, Biopolitics, Roberto esposito

## Abstract

As witnessed over the last year, immunity emerged as one of most highly debated topics in the current Covid-19 pandemic. Countries around the globe have been debating whether herd immunity or lockdown is the best response, as the race continues for the development and rollout of effective vaccines against coronavirus and as the economic costs of implementing strict containment measures are weighed against public health costs. What became evident all the more is that immunity is precisely what bridges between biological life and political life in the current climate, be it in terms of the contentious notion of herd immunity, the geopolitical struggle for vaccines, or the possible emergence of “Covid-elite”, i.e. holders of so-called “immunity passports”. Immunity, as such, is certainly not only a matter of science and biology alone, but is inherently political in the way that pandemics themselves are often highly politicised. Drawing on the work of Roberto Esposito and other literature from the field of biopolitics and immunology, this paper provides a critical examination of the concept of immunity in light of the recent events, highlighting the intersections between the politics of defence and the politics of sacrifice which animate governments’ immunitary responses to the Covid-19 pandemic. The paper ends with a discussion on the forms of solidarity and local initiatives that have been mobilised during the current pandemic and their potential for an affirmative form of biopolitics. Overall, the main aim of this paper is to provide a critical cultural and philosophical analysis of Covid-19 debates and responses and a nuanced account on the biopolitical effects of the current pandemic, highlighting the paradoxical nature of immunity which straddles at once negative practices of defence and sacrifice as well as affirmative forms of community and solidarity beyond state apparatuses.

From life at its most prosaic to its most dramatic, We live unquestionably immunitary lives. (brown 2019: 3)

More than any other recent event, the emergence of Covid-19 pandemic has quickly ushered an unprecedented global crisis with deep social, economic and health implications. Some of the effects can already be felt while others are yet to be seen: from the rising death toll and loss of jobs worldwide to the collapse of relationships under the pressure of lockdown and the psychological impact of confinement, the ramifications are likely to reverberate across societies for many years to come. The severity of the Covid-19 crisis does not only lie in its scope and fast expansion around the world, but also in the radical uncertainty surrounding it: when will it end and how will life look like afterwards? a question that not even the most expert can adequately answer at the moment. For the pandemic, as Yong (2020) argues, is not a hurricane or a wildfire nor is it comparable to 9/11 and similar events. While such disasters are contained in time and space, Covid-19 can linger for years and across the entire globe. The severity of the situation lies also in the lack of preparedness of even the most developed countries and in the way this pandemic has interrupted the “normal” functioning of the everyday, rupturing the very order of neoliberalism and its precarious structures.

Like many previous pandemics, Covid-19 magnifies the intertwinement of biological life and political life whereby the body of the individual and the population is placed all the more at the centre of governmental strategies and interventions for the purpose of ‘defending’ society, as Foucault (2003) previously argued. At the same time, pandemics such as Covid-19 also call into question the relationship between self and other, between the individual and the common, between proximity and distance. Measures of “social distancing” and “self-isolation” have been adopted worldwide so as to contain the spread of the virus and reduce the number of infections. These measures are certainly redefining sociality and responsibility. Despite the risk of loneliness, the “self-as-one” is foregrounded as the safest possible ontology in the current climate, not only in terms of protecting the self from contagion by others, but also for protecting others from the self, as Li (2020) puts it.

At the heart of this logic is the notion of immunity, which emerged as one of the central themes in the medical and political debates concerning coronavirus. Questions have been raised as to whether lockdown or herd immunity is the best policy for dealing with the spread of Covid-19, whether scientists are getting close to developing an effective vaccine that is able to *safely* provide immunity against such an aggressive disease. Although different in essence, these strategies have a common goal: the immunisation of the population via either exposure (herd immunity though mass infection or though inoculation) or protection (lockdown and social distancing). The will to immunity, or what we may call “immunitarianism”, brings the biological and the political even closer as life itself becomes the primary site for enacting policies of defence and pre-emption at the corporeal level while strengthening the state’s control of its subject (through surveillance and tracking for instance). In this article and through a close reading of Roberto Esposito’s work on immunity and other literature from the fields of biopolitics and immunology, I critically examine the concept of immunity not only as a biological concept or a medical term but also as a socio-political metaphor, looking at how current responses to Covid-19 are rooted in the immunitary paradigm of ‘defence’ as well as a politics of ‘sacrifice’, which have various consequences on the way the pandemic is governed and how it is experienced by different groups of society. At the same time, I consider the current situation as an opportunity to revisit some debates on biopolitics and reflect on their merits and limits.

The article is organised into two sections. The first section begins with scientific definitions of immunity and the immune system followed by a philosophical examination of the relationship between immunity and politics as demonstrated by the current responses to Covid-19 pandemic and through the theoretical lens of biopolitics. What is evident is that the adoption of war rhetoric by politicians and healthcare workers when describing the pandemic, the intensification of surveillance technologies and emergency policing powers, the closure of borders and the imposing of lockdowns, all gesture towards a *defensive politics *whereby immunity is articulated in terms of protecting the inside from the outside and defending the body against the disease and those who carry it. Immunity as a biological reality is, nonetheless, irreducible to its defensive function, an argument taken up by some contemporary immunologists and philosophers of science, notably Pradeu and Tauber, who argue for a more ecological, inclusive and communal take on immunity. By bringing such arguments in dialogue with the defensive modality of biopolitics, I highlight the importance of otherness and community in rethinking the concept of immunity as exemplified through the case of convalescent plasma therapy used in the treatment of Covid-19.

The second section of the paper takes, as its point of departure, the controversial concept of herd immunity through mass infection (what is also referred to as “natural herd immunity”) in order to examine another important dimension of the immunitary biopolitics of Covid-19. Here and through a series of examples, I discuss how the pursuit of herd immunity before the development of viable vaccines and the premature reopening of states economies despite known risks is underpinned by a sacrificial logic that rationalises the death of the elderly and the disproportionate exposure of ethnic minorities and precarious workers to the perils of Covid-19. In doing so, this section captures the dialectic at the heart of the politics of the current pandemic whereby defending the population against Covid-19 often involves *sacrificing* certain segments of society and strengthening various forms of inequality and systemic injustices.

But beyond these dual modalities of defence and sacrifice lies the potential for an affirmative form of biopolitics, aspects of which are to be found in the positive community initiatives and collective solidarity that have emerged in response to Covid-19. Invoking the case of Brazil, I discuss how, in the absence of state support and lack of governmental action, community initiatives in the favelas and grass root activism became the major means of combatting the disease and, as such, a powerful example of the possibility of life-affirming politics beyond the state. I warn, however, against the romanticising of community and local initiatives as this runs the risk of shifting health responsibilities to vulnerable groups themselves. For although the possibility of an affirmative biopolitics rests on self-organisation, solidarity and communal initiatives, the state still needs to be held accountable and positioned as the main organiser of healthcare and support.

Overall, the main aim of this paper is to provide a critical cultural and philosophical analysis of Covid-19 debates and responses and a nuanced account on the biopolitical effects of the current pandemic, highlighting the paradoxical nature of immunity which straddles at once negative practices of defence and sacrifice as well as affirmative forms of community and solidarity.

## The defensive politics of immunity

**Fig. 1:**
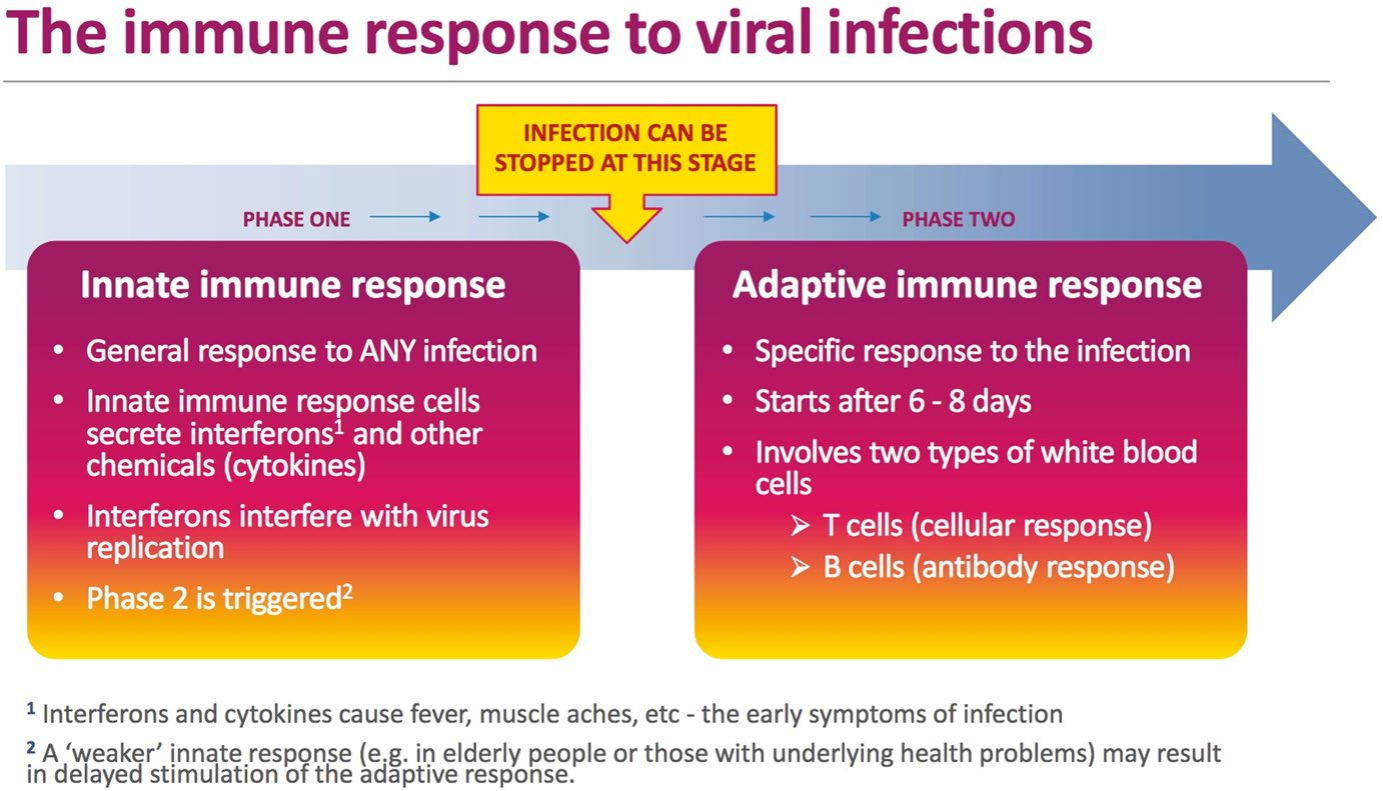
The immune response to viral infections. *Source*: World Health Organization 2020

With or without pandemics, immunity remains an important aspect of everyday life and crucial to one’s survival. Everything alive relies on some kind of effective immune system to neutralise outside threats such as bacteria, viruses, parasites and toxins. A well-functioning system is able to distinguish host tissue from foreign tissue and mount a complex immune response when encountering a pathogen or some other “invaders”. Scientifically, the immune system refers to a collection of chemicals, cells, proteins, tissues and processes that functions to protect the organism from foreign antigens (Marshall et al. 2018). It has two fundamental lines of defence: innate immunity and adaptive immunity (Fig. 1). Innate immunity is the first line of defence to an invading pathogen. It includes the external barriers of the body such as skin and mucous membranes, as well as the activities of immune cells such as macrophages and natural killer cells involved in the detection and destruction of harmful organisms (Marshall et al. 2018; Gavi 2020). Innate immunity has no immunologic memory. It is more general and non-specific, as it cannot recognise the same pathogen should the body be exposed to it in the future. Adaptive immunity, on the other hand, is antigen-specific and has the capacity for memory, which allows the host to mount a rapid immune response in the case of subsequent exposure to the same antigen.^1^ This type of immunity develops as we go through life and get exposed to various microbes and diseases. Adaptive immune responses involve two types of cells: B cells, a type of white blood cell, which ‘help to hunt down invaders circulating in the bloodstream by producing antibodies^2^’ (Gavi 2020) and T cells, which detect and kill cells that have already become infected. ‘T cells also support B cells, helping to control the antibody response’ (ibid.). In addition to these two lines of defence, immunisation through vaccines provides further protection by artificially introducing antigens or attenuated pathogens to a person in such a way that the individual does not become sick but still produces antibodies. Since the body saves copies of the antibodies, it is protected if the same threat appears again later in life. ‘Most vaccines aim to stimulate the development of antibody-producing B cells and the helper T cells which support antibody production, but they can also promote development of killer T cells which target infected cells’ (ibid.). Importantly, in this scientific paradigm, immunity is defined on the basis of ‘self/nonself discrimination’ (Golub and Green 1991). It is, as such, essentially about ‘identity’, the identity of the organism.

Traditionally, conceptions of immunity in science have been bound up with politically invested notions of individuality and identification. According to Tauber (2016a), immunity, as a defence mechanism, is perceived in terms of an attacked self pitted against an alien other. In this understanding, ‘distinct borders confer individuality and immunity is the response to the violation of those boundaries’ (Tauber 2016a). The individual is thus perceived as an isolated, ‘self-contained’ entity requiring protection from the outside. Nevertheless, Tauber points out that immunity extends beyond the confines of an insular individual to include ‘mediation of exchange processes with the environment, where active tolerance allows nutritional assimilation and cooperative symbiotic relationships’ (ibid.). Or as Pradeu (2019a) argues, ‘it is now clear that many foreign entities, such as microbial communities (known as microbiota), are actively tolerated by the immune system rather than eliminated’. This raises the conceptual need to shift from an ‘internalist view’ of self/non-self framework, which sees the individual as autonomous and insular, to a more ‘interactionist view’ whereby an organism is seen as an ecosystem that is constantly interacting with the outside environment (ibid.). With this ecologically informed understanding, both immunity and the individual organism can be regarded as fluid and dependent on the environment, which makes the entire immunity enterprise as an on-going process of establishing and maintaining organismal identity. Immunity in this sense is not merely a matter of science alone but also lends itself to philosophical enquiry into what constitute identity and selfhood. And according to Tauber (2016a), ‘the philosophical challenge of defining immune identity is framed by differing orientations, namely, autonomous individuality versus collective ensemble’.

Such challenge has been taken up by the Italian philosopher Roberto Esposito whose work is centred on the question of immunity in relation to community and organised around concepts derived from biology. In his book *Immunitas *(2011), Esposito provides a lucid and compelling examination of the ways in which immunity acts as a framework for modernity itself, cutting across various domains, discourses and spheres of life. From the outset, the book draws our attention to the fact that each seemingly disparate field in society somehow appeals to the function and logic of immunity in its attempt to respond to threat: ‘[w]hether the danger that lies in wait is a disease threatening the individual body, a violent intrusion into the body politic, or a deviant message entered the body electronic, what remains constant is the place where the threat is located, always on the border between the inside and the outside, between the self and the other, the individual and the common’ (Esposito 2011: (2). Immunity, as such, coalesces with various fields beyond human biology, including law, politics, computer science, borders and so on, in that each of these domains is constantly striving to immunise itself from external danger (both actual and potential) and protect its (imagined) borders from an invading other.

Relatedly, what is also apparent throughout the history of immunity and the contemporary debates around it is that its biological mechanisms are often conceived and described through the language of war. We can see this clearly in the current discussions about coronavirus, which have been dominated by the lexicon of conflict and turmoil. Just as Bush and Blair declared a “war on terror” following the events of 9/11, governments across the world have been declaring a “war on coronavirus”, likening the pandemic to an enemy that must be defeated. In Britain, Boris Johnson created a “War Cabinet” to “battle” the virus while the Queen, in her address, invoked the words of the Vera Lynn wartime song “We will meet again”. In China, Xi Jinping declared a “people’s war” on coronavirus. In the United States, Donald Trump pronounced himself as a “wartime president” fighting against “the Chinese virus”. In France, Emmanuel Macron declared the country at war with an “invisible enemy”. And so on.

Politicians are not the only actors to invoke the war rhetoric in the context of the current pandemic. Doctors and health workers also likened the pandemic to wartime situations and have been portrayed as combatants who are risking their lives at the “frontline” to treat Covid-19 patients. For instance, in an episode of the New York Times’ podcast *The Daily*, the Italian doctor, Fabiano Di Marco, described the influx of Covid-19 patients and lack of medical supply in hospitals as a war. Similarly, in an interview with the Guardian, a nurse from Piacenza hospital in Emilia-Romagna described the challenges in the following way: ‘It’s an experience I would compare to a world war. But it’s a war that isn’t fightable with traditional arms—as we don’t yet know who the enemy is and so it’s difficult to fight. The only weapon we do have to avoid things getting even worse is to stay at home and to respect the rules, to do what they did in China, as this is paying off’ (in Giuffrida 2020). More recently and in discussing the challenges of Covid-19 vaccine rollout, immunologist and Oxford professor John Bell urged the UK government to react to Covid-19 as if to an enemy invading the country: ‘[p]eople have rightly pointed to the Israelis, who have managed to immunise lots of people. You have to view it as if it were a war. The Israelis are good at getting on a war footing—everyone is waiting for the 2am call anyway. Here it is not clear whether it’s a national security issue, but it is. The economic impact is as bad as any war. You might say 100,000 dead is not as bad as a war but it’s still not where you want to be’ (Bell in Otte 2021).

Framing the pandemic in military terms helps communicate the gravity of the situation while justifying state interventions and the demands placed on citizens such as social distancing (Serhan 2020). As Caso (2020) argues, ‘war is a powerful metaphor. It is an effective, immediate and emotive tool to communicate urgency to the general public. It also conveys a sense of struggle and righteousness that can justify exceptional measures.’ Likening Covid-19 pandemic to war is also meant to foster sentiments of solidarity and mobilise collective efforts akin to those witnessed during actual wartime. Yet this war analogy has also been criticised for its somewhat offensive undertone, which seems to suggest that ‘those inflicted by the disease have been called to combat, as if their survival depends on inherent willpower rather than medical, social and economic factors far beyond their control’ (Freedman 2020). The metaphor of war, therefore, displaces responsibility by putting the onus on individuals instead of inviting a critical reflection on the state of healthcare systems and society as a whole as well as the lack of governmental preparedness. As in the words of Gainty (2020), ‘we are seemingly too busy fighting off an attack to consider how the notions of health that we adopted in the twentieth century—along with the subsequent infrastructure of healthcare built up around these beliefs—have produced this bellicose state.’ Invoking war imageries also ends up breeding fear, the impact of which has been apparent in the barren supermarket shelves, the depletion of toilet paper stocks and the overall widespread panic-buying that went on for weeks at the beginning of the pandemic.

Turning again to Esposito here might prove fruitful for understanding further the seemingly close connection between the rhetoric of war and pandemics as well as the political dimension of immunity. There are roughly two key interrelated arguments animating Esposito’s discussion in *Immunitas*. The first being the aporetic nature of immunity, especially acquired immunity through inoculation and the way in which it protects life by precisely incorporating the external threat within the body rather than keeping it at a distance. As Esposito (2011: 9) puts it, immunity ‘can prolong life, but only by continuously giving it a taste of death.’ In other words, within the immunitary paradigm, the body defeats a virus by making it part of itself rather than expelling it outside the organism. This ‘exclusionary inclusion’ (ibid.: 8) makes life possible through its own negation. Threat is carefully incorporated so as to be repelled more effectively. This double logic of immunity informs much of Esposito’s analysis of the concept of politics, a point I shall return to later on. For now, let us take a look at the second overarching argument Esposito makes vis-à-vis immunity and which strikes at the heart of the above-mentioned connection between military analogy and immunity.

Through an etymological journey that traces the genesis of immunity, Esposito reveals that the origins of immunity, as a paradigmatic concept, are to be found in law and politics rather than in biological science and immunology. He takes as his point of departure the Roman law in which certain strata of society were immune from certain civic duties and community obligations and thus, exempt from munus, that is, the obligation of reciprocal gift-giving. He writes, ‘[t]hose who are immune owe nothing to anyone, in terms of both vacatio and excusatio: whether referring to an orginary autonomy or the later release from a previously contracted debt, what counts in defining the concept is exemption from the obligation of the munus, be it personal, fiscal, or civil’ (Esposito 2011: 5). Immunity, in this sense, is not only an exemption but also a privilege that allows the immune individual to turn inwards and remove oneself from participation in the common. Seen in this light, immunity is understood as that which protects the individual from the outside by establishing borders between what is one’s own and what is communal. Yet these borders of differentiation and distanciation are instituted not by the individual herself, who enjoys immunity, but by the law that dictates the norms and functions of becoming immune. So, while immunity may seem to negate community by removing the immune individual from the sphere of the communal and its obligations, it nonetheless remains inextricably linked to the common through the shared law that governs their entire horizon and delimits what is possible in terms of boundaries and exemption. The category of immunity remains, therefore, inseparable from that of community (ibid.: 16) despite the fact that the whole essence of immunity is to separate the individual from the communal, insofar as immunity presupposes community (and its norms) at the same time that it interrupts it and negates it. This paradox inherent in Roman law is, according to Esposito, the primary hallmark of immunity and one that is by no means peaceful or painless.

Taking cue from Simone Weil who identified the Roman paradigm as the root of twentieth-century totalitarianism, Esposito argues that violence and force are fundamental components of law and the protection of legal rights. This follows Weil’s claim that ‘rights are always asserted in a tone of contention; and when this tone is adopted, it must rely on force in the background, or else it will be laughed at … rights are by their nature dependent on force’ (cited in Esposito 2011: 26). As such, just as the body protects itself from threat by making it part of itself through immunisation, so does the law. The protection of rights is applied by force, and law, as Esposito (2011: 29) puts it, is nothing other than ‘violence against violence in order to control violence’. The immunitary function of law consists of saving individuals and communities from violence by mobilising (legitimised) violence so as to manage (prohibited) violence: ‘if violent means such as the police apparatus or even the death penalty are used to exclude violence external to the legitimate order, the legal system works by adopting the same things it aims to protect against’ (ibid.). In other words, through the immunitary mechanism, violence becomes incorporated into the apparatus it seeks to repress. Here Esposito comes very close to Walter Benjamin’s (1996) and Giorgio Agamben’s (1998) arguments that ‘every possible form of “right,” or “common” life, is sacrificed for the mere survival of its bare biological content’ (Esposito 2011: 10). That is to say, in order for life to be preserved and protected, it has to be stripped to its bare status, to the pure nakedness of its biological existence. And again, for life to be preserved in the immunitary sense, ‘something needs to be introduced into it that at least in some aspect negates it to the point of supressing it.’ (ibid.: 33).

In response to the measures mobilised against coronavirus pandemic in Italy (and later on in many other countries), Giorgio Agamben (2020) lamented precisely this sacrificial logic of survival, which reduces life to its bare form in the name of saving it. He argues that ‘the wave of panic that has paralyzed the country obviously shows that our society no longer believes in anything but bare life’ so much so that face to face sociality and civil liberties are readily scarified for the sake of achieving biological security through surveillance and social distancing. This, according to Agamben, ends up normalising the state of emergency and exception to which people have become accustomed for some time. As Owen (2020) elaborates further, ‘governments treat every event as a pretext for the suspension of normal laws. Citizens adapt to the new reality: they defer to the exception and so it becomes the rule. In doing so, some vital element of human life is suppressed or undone.’ This is exacerbated all the more by the rhetoric of war discussed above which legitimises violence by breeding fear and normalising special measures. As Carso (2020) puts it, ‘[f]ear is a tool of control. Frightened people are more likely to accept exceptional measures and limitations to their freedoms.’ We have seen, for instance, that in South Africa, tear gas, whips and rubber bullets have been used to enforce social distancing and discipline citizens found outside their homes without what is deemed by the government as a valid reason (Straits Times 2020). In Philippines, President Rodrigo Duterte threatened to shoot dead those who violate quarantine (Sternlicht 2020). The first reported case was that of a 63-year-old man shot dead at a Covid-19 checkpoint in Nasipit for supposedly violating imposed curfew (Lovett 2020). In China, authorities have been putting pressure on private companies to hand over citizens’ personal data, including location and telecoms data, for anti-epidemic purposes, a trend that is likely to last beyond the pandemic, according to privacy advocates (Zhong 2020). In Israel, a programme was set up involving cell-phone surveillance to locate and send unsolicited text messages to people who might have been exposed to the coronavirus, ordering them to self-isolate (Estrin 2020). Countries in the West have also been deploying various surveillance mechanisms to enforce lockdown. In the UK, police have been using drones to ‘shame’ people into staying indoors (Bienkov 2020), while the UK ‘Coronavirus Act 2020′ extends police powers of detention and removes certain safeguards vis-à-vis state surveillance. Various tracking apps have been deployed worldwide for ‘contact tracing’ in order to contain the spread of the disease through the identification of people who might have come in contact with someone confirmed to have the coronavirus (Collins 2020). In Hungary, Prime Minister Viktor Orbán used Covid-19 emergency law to seize unlimited power and extend his rule indefinitely. And the list goes on.

Such measures are part of the immunitary paradigm Esposito speaks of, which seeks to immunise life against threat by injecting various mechanisms into it that are, at times, violent, symbolically at least if not also materially. More recently, he wrote that the governmental responses to the coronavirus pandemic are symptomatic of ‘a veritable immunization syndrome that has long characterized the new biopolitical regime’ (Esposito 2020). This is a ‘defensive biopolitics’, a form of politics that manages life and the living in a way that shifts from ordinary democratic processes to emergency measures, bringing political procedures of democratic regimes into conformity with those of authoritarian states (ibid.). Though, as Christaens (2020) argues, the current state of emergency declared by many countries is perhaps more symptomatic of ‘institutional breakdown’ resulting of years of budget cuts in healthcare and the neoliberalisation of almost every sphere of life, rather than ‘a totalitarian ploy’ to dominate the population. Still, the responses to the pandemic are increasingly blurring the lines between democracy and totalitarianism, between care and control as demonstrated through the intensification of monitoring and control witnessed over the last year.

For Agamben, it is not surprising that the language of war is invoked when speaking of the virus in as much as the emergency measures, adopted as a response, create warlike conditions, such as curfew and enhanced surveillance, casting the virus as ‘an invisible enemy that can lurk in every other person’ (Agamben 2020). This, for Agamben, is tantamount to a ‘civil war’ where everyone is seen as a vector of contagion and where the enemy is not outside, but ‘within us’—just as in the ‘war on terror’ everyone became a potential terrorist in the eyes of governments. While Agamben has been accused of being a “coronavirus denialist” (Christaens 2020) and a “paranoid” (Berg 2020), “misguided sage” (Owen 2020), I would argue that his warnings are, nonetheless, valid and warrant to be taken seriously. We have seen time and again throughout the recent decades how practices and mechanisms that are initially designed for specific exceptional circumstances and particular spaces end up becoming routine and widespread across the entire fabric of society. One example is to do with the application of biometric technology. The initial social and political use of biometrics was limited to exceptional spaces and extreme cases, such as detention centres and crime investigations. Following 9/11 and other events, biometrics became more widely used so much so that it is now embedded in everyday products and services. We use biometric fingerprints and facial recognition to unlock our phones or log into our bank accounts; we use MobilePay to purchase our morning coffee; we use fitness trackers, such as Fitbit, to log our daily exercise; voice recognition to interact with virtual assistants such as Amazon’s Alexa and Google Assistant and so on. Technologies that would have seemed intrusive a few years ago are so commonplace today that it can seem paranoid to resist them (Lynskey 2019). Some years ago, Giorgio Agamben, warned against this spill-over and normalisation of biometric technology, a process he referred to as “bio-political tattooing” (Agamben 2004). Agamben famously cancelled a course he was scheduled to teach at New York University refusing to travel to the United States where new security measures requiring foreign nationals to submit fingerprints had been introduced. What Agamben took issue with was how the body itself had become both the object and the means of control and intervention; his main objection being that the “bio-political tattooing”, imposed by the U.S., could very well be the precursor of ‘acceptable’, ‘normal’ practices of identity registration and verification in the future. Agamben’s prophecy seems to have come true: many countries have, since then, adopted compulsory biometric border identification and use biometrics in commercial spheres, too. And with the current technologies being developed and deployed to trace contact and track the whereabouts of people as a surveillance mechanism against the spread of coronavirus, we are likely to see a similar function creep and repurposing that may well outlive the pandemic itself.

Circling back to the curious genealogy of immunity undertaken by Esposito, let us consider for a moment the movement of immunity from the juridico-political domain to the biomedical science and immunology’s appropriation of the politico-legal language of war and death that has marked the notion of immunity since its inception. In a chapter dedicated to the concept of biopolitics, Esposito (2011: 121) asserts that ‘the point of intersection between political knowledge and medical knowledge is the common problem of preserving the body.’ Therefore, the crossing of political and legal concepts into biomedicine and biology (and vice versa) is not too surprising. For, the body is, after all, both ‘the instrument and terrain’ of all forms of immunitary preservation and the ‘frontline, both symbolic and material, in life’s battle against death’ (ibid.: 113). As such, Esposito believes that ‘even the simple figurative superimposition of the biomedical on top of the legal-political language in the representation of the body implicitly points to the question of its immunity’ (ibid.: 122). Drawing on Foucault, he goes on to add that the immune framework developed from the eighteenth century onward involved a shift from sovereign power (power to take life or let live) to biopower (power to make live or let die), which in turn, entailed the coming together of therapeutic practice and political order: ‘to become the object of “care,” life had to be separated off and closed up inside progressively desocialized spaces that were meant to immunize it’ (Esposito 2011: 140). This means that the immunisation of life involved the spatial separation of neighbourhoods and the erection of borders between zones and communities, which went hand in hand with medical developments including the discovery of antibiotics against infectious diseases. One example is that of nineteenth-century English cities which, as a result of the cholera epidemic of 1832, witnessed a separation between rich and poor neighbourhoods together with the enforcement of compulsory sanitary measures (ibid.). In this sense, the political and the medical spheres are inter-implicated in their pursuit to immunise life through the body of the individual and the population.

The last chapter of *Immunitas* provides a clearer sense and a more explicit account of the migration of the jurdicio-political discourses around immunity into the medical field and immunology. Through a discursive engagement with the writings of famous immunologists, such as Frank Macferlane Burnet and Jan Klein, Esposito demonstrates how the political metaphors of warfare and defence permeated scientific discourses of immunity during the twentieth century echoing ‘the armamentarium of the clashes against “invading hordes”’ and the identitarian distinction between self and non-self (Esposito 2011: 154–155). For instance, recalling John Dwyer’s *The Body at War: The Miracle of the Immune System* (1988) and Lennart Nilsson’s *The Body Victorious* (1987), Esposito comments on how the semantics of these medical books is littered with military metaphors such as the detection of the enemy, the activation of the defence lines, the launch of counterattacks, the use of ammunition, the capture of opponents, the clearing away of casualties from the field and so on. He writes, ‘[a]ll the phases of the immune battle between “self” and “non-self” are reconstructed with such a wealth of details one begins to wonder if these are medical accounts explained using military images, or military strategy books illustrated by medical metaphors’ (Esposito 2011: 156). What results out of this seemingly symbiotic relationship between medical discourse and military rhetoric is that politics—with its obsessive attention to the boundaries of identity, its phobic fear of infection and its continuous erection of new defensive borders—gains more strength and authority thanks to the legitimacy of medicine and scientific endorsement (ibid.: 154) (we have seen the worst actualisation of this through the case of Nazism).

Esposito is not alone in locating the genesis of immunity in the sphere of law and politics rather than biology and medicine. Ed Cohen, for in instance, also provides a thorough examination of the etymology of immunity and its subsequent appropriation by immunologists. In his book, *A Body Worth Defending* (2009), Cohen argues that ‘for nearly two thousand years, immunity, a legal concept first conjured in ancient Rome, has functioned almost exclusively as a political and juridical term’ (Cohen 2009: 3) before being imported by medicine and applied to ‘vital contexts’ (ibid.: 274). To this he adds that the concept of ‘self-defence’ also originated as a political idea, ‘emerging only 350 years ago in the course of the English Civil War, when Thomas Hobbes defines it as the first ‘natural right.’ One hundred and twenty five years ago, biomedicine fuses these two […] political ideas [immunity and self-defence] into one, creating ‘immunity-as-defense.’ It then transplants this new biopolitical hybrid into the living human body. We have not been the same since’ (ibid.: 3). He explains that, just as in Roman law immunity meant an exemption from certain obligations (as discussed above), in the field of immunology, immunity came to mean an exemption from the experience of certain diseases and infections. As such, biomedical interpretations of immunity seem to have faithfully preserved the original political meaning of immunity. The central and recurring argument underpinning Cohen’s book is that immunity has ‘no “natural” significance whatsoever’ (ibid.: 45) and that it is primarily a political idea that has been applied to biomedicine to describe actual biological phenomena—even if they do not necessarily resemble the original meaning of immunity found in its politico-legal heritage. He writes.

the language of friend and enemy in no way derives from the matter of the world; it does not describe the unfolding of biochemical processes according to immutable natural laws; it does not constitute an unmediated representation of an essential physical truth; rather, the trope of friend and enemy has circumscribed Western politics since Aristotle. In fact, it has provided a canonical framework for defining “the political” as such ever since there first was a polis. More important for my purposes, it has also underwritten the bioscientific investment in immunity as a biological form of self-defense for the last one hundred years. (Cohen 2009: 277).

Like Esposito, Cohen regards the emergence of biological immunity in history as a concrete example of biopolitics insofar as it ‘makes the body modern’ (ibid.: 10) rendering it amenable to medical and political interventions. However, this seemingly neat historicisation of immunity is not without its problems. There is, first of all, a degree of essentialism in Cohen’s argument with regard to the supposed immutability of nature. This argument also revives a dichotomous thinking about nature/culture, politics/science, which has been challenged by many disciplines, including feminism, media studies, Science and Technology Studies (STS) and so on, in favour of a more integrative approach that foregrounds the entanglement and co-evolving of these categories. As Jamieson (2016: 124) argues, Cohen’s postulation (and in some way, Esposito’s too) assumes a prior separation between politics and biology and the existence of a natural, pre-political state, whereas, ontologically, these domains are not necessarily separate from each other (ibid.: 119).^3^

Furthermore, it is important to point out that, though historically immunity has been primarily understood and conceptualised in terms of defence as discussed thus far through Esposito’s and Cohen’s analyses, the biological function of the immune system itself is by no means reducible to its defence activity. Thomas Pradeu (2019b), for instance, argues for an extended view of immunity that goes beyond the discourse of immunity-as-defence. Referring to recent research in the field of immunology, he states that immune processes play a crucial role in a wide variety of physiological activities and internal housekeeping, including development, tissue repair and maintenance, clearance of dead cells, metabolism and the overall functioning of the nervous system and cognition (Pradeu 2019b: 9). He goes on to state that ‘[p]erhaps one could even consider that the very division of these processes into such categories as “defense,” “repair,” and “development” reflects more the way we, as investigators, address questions about bodily systems (and divide such processes into convenient categories) than real differences in nature’ (ibid.: 10). As such and given its eclectic functions, it is somewhat challenging to provide a singular, precise and consensual definition of immunity, according to Pradeu, to the extent that ‘[f]ocusing on defense against pathogens would be too narrow. On the other hand, extending the definition of immunity so far as the overall physiological regulation of the body […] would be so broad that it might cease to be scientifically fruitful, as almost everything in biology could be said to be immunological, at least to some degree’ (Pradeu 2019b: 13).

Pradeu’s quasi solution to the quagmire of defining immunity beyond defence is to say that ‘immunity is one of the main devices insuring the cohesion of the organism and the delineation of its boundaries’ (ibid.). This view takes Pradeu straight to the debate on biological individuality and identity and their relation to immunity. It does so in a move that is reminiscent of Tauber’s above-mentioned arguments regarding the need to go beyond the self/non-self division that underpins traditional conceptions of immunity within immunology and consider other relevant aspects such as immunological tolerance, symbiosis and autoimmunity, all of which demonstrate how ‘self’ components can trigger immune responses while ‘non-self’ components can be actively tolerated by the immune system (ibid.: 20). This view challenges orthodox framings of immunity while calling for a more relational and ecological take on immunity. As put by Tauber (2016b: 209),

When older definitions of immunity shift from the original defensive formulation in service to an autonomous entity to one that embraces an ecological orientation, agency assumes a relational structure and new designs of identity are invoked […] That conceptual re-orientation has resonance beyond the laboratory and constitutes a rich example of science and society.

Tauber goes on to argue that the self/non-self distinction which informs immune discrimination is in fact a projection of social prejudice which casts contested social boundaries as “natural” rather than socially constructed:

Just as Social Darwinians promoted "the survival of the fittest" as a trope to capture the social essence of America a century ago, today "immune reaction" putatively functions in parallel fashion to that earlier projection […] The warfare metaphors—"attack," "defense," "invaders"—so prevalent in immunology's lexicon, dramatically illustrate this construction, both in terms of the self/nonself dichotomy, as well as the privileged standing of individuality over the commune […] Accordingly, immunology provides a political metaphor for American culture marked by atomistic individuality […] An ecologically inspired research program challenges immunity conceived solely in a defensive format. (ibid.: 211–212).

But despite the myriad attempts by some contemporary immunologists as well as sociologists and philosophers of science to redefine immunity and broaden its scope to subsume relational understandings of cooperative rather than individualistic nature, it remains that the self/non-self trope, with its rhetoric of defence, still represents, as Pradeu (2019b: 17) asserts, ‘the dominant, if often implicit, framework in which immunologists conceive how the immune system works’. This, according to Tauber, has to do with a much larger ‘drama’ revolving around the very values of autonomy and freedom that structure Western societies and their liberal precepts of individuality. For, ‘despite efforts of the most influential twentieth-century philosophers to revamp the modernist conception of mind and personal identity […], the basic appeal of human autonomy dominant during the modern period is not easily dislodged’ (Tauber 2016b: 219). As such, just as traditional notions of identity and individuality have somewhat resisted displacement despite postmodern tireless attempts to decentre and deconstruct the Western subject, so too have traditional notions of immunity and their overemphasis on organismic borders and their defence, despite that, ontologically, immunity’s functions are far more varied.

We can see the persistence of the defensive self/non-self framework unfolding in the case of Covid-19. In addition to the above-discussed war rhetoric adopted by various politicians in their response to coronavirus outbreak and the ensuing individualising measures such as social distancing and quarantine, scientists themselves have repetitively invoked the language of defence in their discussions and clinical studies of Covid-19. For instance, a recent article from *Nature Reviews Immunology* by Osier et al. (2020) brought together various international immunology societies to discuss how their field responded to Covid-19 pandemic. In most responses, immunity was articulated in terms of defence and self-protection as if these were the default vocabulary for referring to immunity. This is more so the case when scientists attempt to communicate the debate on immunity and Covid-19 to the general public (see for example the Public Information section of the British Society for Immunology’s website). The discourse of immunity-as-defence is, in fact, so normalised that even when explaining immunity and life processes to children, there is a tendency to fall back on the war analogy and assume that children are familiar with the metaphors of conflict.^4^ For instance, in an episode of the New York Times’s podcast *The Daily*, guest scientist, Carl Zimmer, answered questions from America children about Covid-19 and immunity in the following way:

We have something in our bodies called the immune system, which is how we defend ourselves against viruses and bacteria and other bad things. And so you’ve got these immune cells that are wandering around inside your lungs, just making sure everything’s OK. And if they come across viruses or infected cells, they start talking to each other and say, we have got to start a war. And so the immune system can actually make little weapons called antibodies that can fight the virus and clear it out. And so there’s this big battle that starts going on inside of our bodies. In a lot of cases, when people get Covid-19, they feel a fever. Well, that’s actually the immune system kind of heating up the body. (Zimmer in New York Times 2020).^5^

Nevertheless, there is now a wider recognition of the ‘communal’ aspect of immunity, not only in terms of herd immunity, a debate I shall return to in the next section of the paper, but also the role of ‘convalescent plasma therapy’ in treating Covid-19 patients. As explained in a recent scientific article by Ferreira and Mostajo-Radji (2020: 1), this therapy involves ‘[c]ollecting plasma, the liquid component of blood, from someone who has recently recovered from COVID-19 and infusing it into someone with an ongoing infection [to] confer the plasma recipient with antibodies to combat the virus.’ The idea behind this type of therapy is that, in the aftermath of an infection, plasma is often rich in antibodies against the pathogen which caused it and can therefore hasten the recovery of those currently suffering from the disease (Gavi 2020). Unlike the long-lasting immune memory produced by a vaccine, convalescent plasma therapy could offer immediate transient treatment. While further evidence and trials are still needed to determine the efficacy of such technique, this type of therapy does, nonetheless, demonstrates how self-immunity can be co-communal in the sense that the ‘other’ becomes the vehicle through which one can acquire immunity, if only in a fleeting sense. Same could be said of the important role of volunteers in the development and testing of new vaccines, a process which often involves exposing healthy volunteers to a pathogen to learn more about the disease it causes and test vaccines quickly (McPartlin et al. 2020). Vaccines, in this sense, rely on altruistic risks that others are willing to undertake so as to contribute to the protection of the rest of the population.

As such, rethinking immunity beyond borders and defence, beyond self/non-self and in terms of a more relational and cooperative framework, as Tauber and Pradeu argue, is certainly an important epistemological (and political) task for both scientists and philosophers. Although not so apparent at first sight, there is indeed an affinity between the arguments put forward by Tauber, Pradeu and other contemporary philosophers of immunology and the arguments articulated within the biopolitical approach of Esposito and Cohen. Despite adopting different vocabularies and epistemological lenses, they all consider the many ways immunity and its reactions could be inclusive of the outside and maintain a relationship with the other that is not based on rejection and defence but on inclusivity and cooperation. They thereby emphasise the role of community and co-existence for challenging and rethinking the traditional meanings and functions of immunity, both biologically and in the socio-political sense.

So far, we have examined the defensive politics of immunity as manifested through the case of the current Covid-19 pandemic whereby the virus is portrayed as an invading enemy that threatens individuals and nations. What follows from such figuration is the mobilisation and legitimisation of various social measures and surveillance techniques so as to contain the disease and defend the population. Nevertheless, defending the population, as Foucault (2003) reminds us, also often involves sacrificing segments of it, a logic that is at the heart of modern politics and its biopower; the power to ‘make live and let die’ (Foucault 2003), as discussed earlier. As such, to fully understand the politics of Covid-19 and its ramifications, it is not enough to look at its defensive aspect only. One should also consider the other side of the equation, that is to say, the ‘letting die’ aspect. To this end, the next section of the paper will be discussing instances whereby certain political responses to Covid-19 have involved the willingness to sacrifice certain groups for the (economic) survival of the majority and in some cases, a total abandonment of the population by its government, as with the example of Brazil. In doing so, the next section turns the focus towards the contentious concept of ‘natural herd immunity’ (or herd immunity through mass infection) which provides a way into understanding the sacrificial aspects of Covid-19 politics and how certain groups have been disproportionately affected by the pandemic through the very biopower of ‘letting die while making live’ that is inherent within the logic of contemporary forms of governance.

## The sacrificial politics of natural herd immunity

The term ‘herd immunity’, also called ‘community immunity’ (Healthline 2020), refers to a state whereby so many people in a population become immune to an infectious disease that it stops the disease from spreading by providing indirect protection to those who are not immune to the disease. As Fine (1993: 265) puts it, herd immunity ‘has a special aura in its implication of an extension of the protection imparted by an immunization program beyond vaccinated to unvaccinated individuals and in its apparent provision of a means to eliminate totally some infectious diseases.’ Essentially, herd immunity is about mathematics. Its theory rests on the basic ‘reproduction rate’, that is to say, how many new infections each case will produce. For instance, a reproduction rate with a value of 1 means that one infected person can pass it to at least one other person. And the higher this number, the more infections could be generated from that one case. Ultimately, to end the spread of the disease, the reproduction rate needs to be below 1 (Basu 2020). Herd immunity, as such, aims to ‘lower the number of susceptible people to the point where the reproduction rate drops below 1 and the spread of infection stops’ (ibid.). Often, herd immunity is invoked in the context of disease eradication programmes based on vaccination through which ‘the proportion of immune individuals is so high that the number of *susceptibles *is below the epidemic threshold’ (Fine 1993: 267–270, italics in original). Infections such as measles, rubella, mumps, chickenpox and polio are examples of diseases that were once common but are now rare, as vaccines helped establishing herd immunity (D’Souza and Dowdy 2020). However, vaccination is not the only means of achieving herd immunity. Exposure of a large proportion of the population to diseases and subsequent infections are also considered, sometimes, as a way of achieving immunity ‘naturally’ by acquiring antibodies through infection on a mass-scale (as mentioned earlier, this approach is referred to as ‘natural herd immunity’). But as D’Souza and Dowdy (2000) argue, natural herd immunity approach might be reasonable for less severe diseases, but in the case of more severe diseases, such as Covid-19, there is a much higher risk of complications and even death. Vulnerable groups such as the elderly and people with pre-existing health conditions and weakened immune systems are, particularly, at a heightened risk.

Following the rapid spread of Covid-19, the concept of natural herd immunity emerged as a subject of much contention and debates in various countries around the world as the economic costs of implementing stringent containment measures are weighed against public health costs. For instance, Trump famously tweeted on March 22nd 2020: ‘we cannot let the cure be worse than the problem itself’ referring to what, in neoliberal terms, is seen as the danger of disrupting the market for the sake of health. While in the UK, Dominic Cummings’s earlier stance was reportedly summarised as ‘protect the economy and if that means some pensioners die, too bad’ (in Walker 2020). And in Sweden, the government praised its controversial strategy of imposing minimal restrictions, declaring the capital as being on course to reach herd immunity by May 2020—though the cost has been the high death toll (especially of the elderly) compared to other neighbouring countries and natural herd immunity failed to materialise, according to more recent reports (see Orlowski and Goldsmith 2020).

Scientists have warned that, without a viable vaccine, natural herd immunity strategy poses a real danger to public health (Basu 2020). According to Rossman (2002), an expert in virology, achieving natural herd immunity in the UK would require more than 47 million people to be infected with an estimated 2.3% fatality rate and a 19% rate of severe disease. This means that the pursuit of natural herd immunity in the UK, without the availability of an effective vaccine, could result in more than a million people dying and a further eight million needing critical care. Rossman goes on to assert that ‘even if we manage to protect the most vulnerable people (though no discussion is provided on how this will be done or for how long) the fatality rate for the otherwise healthy portion of the population may still be 0.5% or higher. This means that even in this unlikely “best case” scenario we would still be looking at more than 236,000 deaths’ (Rossman 2020).

It is no wonder then that natural herd immunity has become the subject of much controversy. The willingness of some countries to accept the death of the elderly and the sick as an alternative to economic damage caused by stricter containment measures has led anthropologists, Laterza and Romer (2020), to regard natural herd immunity strategy as a form of “market eugenics” whereby the weak and the sick are scarified for the survival of the “herd” and the economy (Laterza and Romer 2020). Historically, the term ‘eugenics’ has been associated with selective breeding programmes, forced sterilisation, medical experiments, mass extermination and concentration camps. It served as a branch of scientific state management throughout the twentieth century, particularly in Britain, the United States and Scandinavian countries, with the dual aim of encouraging people of good health to procreate while preventing others deemed unfit from reproducing in order to end certain diseases and disabilities. Eugenics, as such and even in its more recent ‘liberal’ incarnations (parental choice, pre-implantation screening, prenatal testing, genetic editing and enhancement, etc.), has always implied favouring the survival of the fittest; those who are considered by the state and science as physically healthy and socially fit.

Beyond these historical associations and in the context of the current pandemic, ‘it is the very argument that the economy is more important than people's health that is based on a eugenic logic. Instead of the ethnos and the nation, we have the market that rules supreme over people's lives and is given the power of life and death over its subjects’ (ibid.). In its intersection with politics and economics, immunity thus becomes more than a matter of defending society as a whole from the spread of disease, but also a matter of exposing some to danger for the sake of the survival of the economy. Immunity, as Brown (2009: 3) puts it, ‘makes life both possible and perilous’. This aporetic aspect of immunity together with the logic of market eugenics end up casting the elderly and those with pre-exiting health conditions as the “others” of the pandemic (Lateza and Romer 2020) whose sacrifice is made to seem almost inevitable if the sovereign market is to survive. Epidemiological research into the patterns of mortality among age groups has already shown that older adults have been disproportionally affected by Covid-19 pandemic (see Levin et al. 2020; O’Driscoll et al. 2020; Kang and Jung 2020). According to a statistical study conducted by Yanez and his colleagues, of the 178,568 COVID-19 deaths reported in a six-week sample period from a total population of approximately 2.4 billion people from 16 countries, 153,923 deaths (86.2%) were in individuals age 65 years or older (Yanez et al. 2020). In Britain, ‘Covid-19 was the leading cause of death for male care home residents, accounting for a third of all deaths and the second most-common cause of death for female residents, after dementia and Alzheimer's disease’ (BBC 2020). Nursing homes in other countries have also reported “excess deaths” among their residents since the outbreak of the novel coronavirus, a situation that has been exacerbated by the lack of protective equipment and testing for both residents and care workers (Ortiz 2020).

As politicians around the world were getting anxious to restart the economy after lockdown, the death of the elderly was often implicitly, and at times explicitly, cast as a trade-off, a price to be paid for saving the economy (Pittis 2020; Bass 2020). We can recall here the lieutenant governor of Texas, Dan Patrick, who told Fox News in March 2020 that ‘he would rather die than see public health measures damage the US economy and that he believed “lots of grandparents” across the country would agree with him’ (Beckett 2020). He praised Donald Trump’s focus on the economy, stating that he does not want ‘the whole country to be sacrificed’ and, as someone who was turning 70, ‘he was in the high-risk group, but he was willing to give up his life for his six grandchildren’ (in ibid.). But as the 90-year-old artist, Varda Yoran (2020), wrote recently, just because someone is old, it does not mean they are ready to die or they are disposable. No limit should be set on when a person’s life is no longer valuable, she argues.

Such narratives have also been playing out in the media in the form of a generational battle between ‘medically imperilled boomers and economically imperilled millennials’ (Lennard 2020), between “reckless millennials” throwing “coronavirus parties” to intentionally infect each other (Crump 2020) and ‘get over with it’ and “selfish boomers” believed to have long wielded their spending power and voting rights to effectively bend ‘the gravity of politics towards themselves and their needs’ (Lewis 2019). This seemingly endless cross-generational war between boomers and millennials has been raging way before the coronavirus pandemic. As Lewis (2019) postulates, ‘[bo]th sides claim that the other is being condescending. Some Boomers argue that the word itself has become a cover for ageism; Millennials roll their eyes and reply with the phrase that has come to encapsulate their weariness: “Okay, Boomer.”’ To explain the basis of this cultural war, Lewis invokes the British politician, David Willetts, who argues in his book, *The Pinch* (2010), that

British “baby boomers”—the generation born in the 20 years after the Second World War— “took their children’s futures.” They bought cheap houses, which have since rocketed in value. Their standard of living rose throughout their working lives. They retired with solid state-funded pensions and sometimes generous private ones, too. Their Millennial children, by contrast, were having a tougher time—struggling to buy a house, watching their salaries stagnate and looking ahead to a much less comfortable retirement. (Lewis 2019).

Not long after the outbreak of coronavirus, a post appeared on Twitter gleefully referring to the virus as “Boomer Remover”. This morbid, and at the same time ‘chillingly astute’ (Sparks 2020), term picked up on the fact that Covid-19 tends to severely affect people over 60 mostly, including the boomer generation (though no one is spared the risk of catching the virus). The term has since become a trending meme among teenagers and ‘a battleground for generational warfare on social media, frequently couched as a natural consequence of how the Baby Boomer generation treated the planet or approached politics’ (Whalen 2020). Others consider the term as a reflection of ‘the general lack of empathy in society these days’ (Foroohar 2020). But as Lennard (2020) points out, the generational analysis and its underlying divide ignores the divisions of class and racialised inequality that Covid-19 has exposed and, in many ways, exacerbated.

Few months after the coronavirus outbreak, reports were emerging about how individuals from Black, Asian and Minority Ethnic (BAME) groups were dying in disproportionate numbers. A study conducted in the UK in April 2020 by the Intensive Care National Audit and Research Centre revealed that, despite making up only 13% of the population, BAME Covid-19 patients account for a third of those critically ill in hospitals (Booth 2020). The reasons behind such disparity are varied and often intertwined, ranging from the prevalence of pre-existing health conditions among BAME communities to socioeconomic factors affecting the living conditions of these groups. For instance, studies in health trends show that people from ethnic minority backgrounds live in more deprived areas and overcrowded accommodation or multi-generational households (Khunti in ibid.). According to government figures, 30% of the UK Bangladeshi population are considered to live in overcrowded housing compared to 2% of the white British population. 15% of black African people also live in cramped housing (Booth 2020). People from BAME backgrounds are also more likely to work in low-paid precarious jobs that do not offer the option of working safely from home during the lockdown. These factors add to the fact that ethnic minority groups have worse health than average, lower life expectancy and a greater prevalence of serious health conditions such as cardiovascular diseases, diabetes and asthma (Khan 2020).

Moreover, BAME workers are disproportionately represented in frontline roles and emergency occupations—whether in terms of nursing the sick in NHS hospitals, taking care of the elderly in care homes, stocking shelves in supermarkets, distributing food to households or keeping public transport operational, BAME workers are having to go out and work risking higher exposure to the virus when the rest are sheltered in their homes (Sullivan 2020). According to Khan (2020), people from BAME backgrounds make up 40% of NHS doctors and 20% of nurses. And, in London, 67% of adult social care workers are drawn from BAME groups. Also, more than a quarter of transport workers operating London buses and underground transport are from BAME backgrounds (Booth 2020). Respondents to a survey conducted by ITV News on BAME deaths among NHS staff complained about unfair treatment and the disproportionately high deployment of BAME staff in “Covid-19 areas” of the hospitals. One respondent stated that ‘all BAME nurses [have been] allocated to red wards and my white colleagues [are] constantly in green wards’ (in ITV News 2020). A report by Public Health England (2020) also argues that ‘[h]istoric racism and poorer experiences of healthcare or at work may mean that individuals in BAME groups are less likely to seek care when needed or as NHS staff are less likely to speak up when they have concerns about Personal Protective Equipment (PPE) or risk.’ So, the trenchant message here is that the current pandemic does not affect everyone in the same way nor is its impact felt equally. This is not only in a biological sense or in terms of Covid-19′s health implications for old versus young generations, but also in terms of the familiar class and racial aspects that are deeply entrenched in society and in the exploitative workings of capitalism and its market eugenics.

This is also evident in the case of seasonal farm workers. At the time of state-imposed travel restrictions, thousands of workers were being flown from Eastern Europe to the UK to pick farm produce and save the harvest from rotting. After a “feed the nation” government appeal failed to recruit enough British workers, special charter flights were organised to fly Romanian seasonal workers to Britain (O’Carroll 2020). A similar trend was observed in Germany and other European countries. Following the death of a 57 years old Romanian worker in Germany from Covid-19-related illness, concerns were raised about the exploitative working conditions faced by seasonal harvest workers (Rombach and Arens 2020). From employment contracts which allow employers to extend the working day to fourteen hours and, if necessary, seven days a week, to the lack of protective masks, short quarantine and crowded living conditions, many workers are left exposed to high-risk working and living situations (Salyga 2020). In Spain where thousands of Moroccans, mostly women, were brought from poor rural parts of Morocco to pick fruits in Spanish fields, many were left stranded without income nor suitable accommodation when the harvest season ended in May and after Morocco closed its border to contain the spread of coronavirus (Maestro 2020). What all these cases illustrate is that the safety of quarantine is not an option for everyone as some people are expected, and even forced by circumstances, to take more risks than others during the pandemic so that the rest remain fed and cared for. As incisively argued by Salyga (2020), the phase of ‘privileged self-isolation’ coincided with the arrival of armies of seasonal workers and the exploitation of wage differentials across Europe and beyond.

As such and in pursuit of immunity, the politics of defence ends up working in tandem with the politics of sacrifice strengthening and producing various forms of inequality, precarity and systemic injustices, with fatal consequences at times. And as the world now faces the monumental challenge of rolling out what has been described as ‘the largest, most complex vaccination programme in history’ (Redgrave et al. 2020), there are concerns that the delivery of Covid-19 vaccine programmes could lead to widening health and economic inequalities if adequate steps are not taken, especially in the context of initial supply constraints and the prioritisation of specific population groups. Andrea Taylor, a Duke University researcher who is studying Covid-19 vaccine contracts, argued that ‘the high-income countries have gotten to the front of the line and cleared the shelves’ (in Twohey et al. 2020). So, while the world’s richest countries have reserved enough vaccine doses to immunise their populations multiple times over, many poor countries will only be able to immunise a small percentage of their population. As indicated in a recent article by Burgess et al. (2020), ‘[e]stimates as of Dec 2, 2020, suggest direct purchase agreements have allowed high-income countries to secure nearly 4 billion confirmed COVID-19 vaccine doses, compared with 2·7 billion secured by upper and lower middle-income countries. Without such agreements, low-income countries would probably have to rely on COVAX, which would achieve only 20% vaccination coverage’—which is not enough for achieving herd immunity through vaccination. All of this could leave behind populations and communities from already disadvantaged areas, reinforcing health inequalities and worsening the impact of the disease. While such challenges continue to unfold, the global elite has been looking to find ways to beat the vaccine queue which, so far, has been largely controlled by national governments. As vividly described by Rickett (2021), exclusive travel and lifestyle services such as, Knightsbridge Circle, are pioneering luxury travel based on Covid-19 vaccines, enabling their rich customers to fly to Dubai or Abu Dhabi in the UAE or to India where they can receive the Covid-19 vaccine of their choice before taking sightseeing trips and hitting the beach in the wait for the second shot. A spokesperson from the Dubai-based health insurer, Eternity Medicine Institute, reported that the vaccine being provided to wealthy tourists is coming from stocks intended for Emirati residents (in Rickett 2021). Such disconcerting trend represents the equivalent of “red carpet access” to Covid-19 vaccines and it is happening at a time when many frontline workers and vulnerable groups have yet to be vaccinated.

In many ways, then, what the current crisis highlights is ‘the inevitability of a struggle over how we are governed, a struggle, that is, over the ways that politics and biology will interface and be configured’ (Short 2020). This is at once a class struggle in the Marxist sense and a biopolitical struggle in the Agambanian sense whereby what is at stake is life itself and the need for an ethics of living in which no life can be destroyed or sacrificed in favour of another, as Esposito (2008: 194) asserts. Beyond the ‘barbarism’ of capitalist market eugenics there lies, according to Žižek (2020: 70), an opportunity to reinvent communism, ‘not as an obscure dream but simply as a name for what is already going on […;] “disaster communism” as an antidote to disaster capitalism’ (ibid.: 103). The provisional suspension of market mechanisms and the attendant interruption of consumerism, the governmental support for the newly unemployed, the mobilisation of local communities and the creation of new bonds of solidarity, the rise of international cooperation and mutual aid networks, as witnessed over last year, are all, for Žižek, hopeful signposts for an alternative politics to come. In his reflections on the Covid-19 pandemic, Alain Badiou (2020) argues that ‘we must take advantage of this epidemic interlude and even of the—entirely necessary—isolation, to work on new figures of politics, on the project of new political sites and on the trans-national progress of a third stage of communism.’ Similarly, Arundhati Roy (2020) believes that coronavirus is ‘a portal, a gateway between one world and the next’ since, historically, ‘pandemics have forced humans to break with the past and imagine their world anew’.

While it might seem naïve to presume that a total revaluation of values will ensue as a result of the pandemic (let alone a sequel, or an entirely new form, of communism), we are nonetheless witnessing many instances of life-affirming solidarity. As Kaye (in Coffey 2020) points out, ‘[e]ver since the pandemic started, we’ve seen mutual aid groups springing out of the soil overnight’ where the young and able-bodied are providing assistance to their infirm and elderly neighbours, be it in terms of picking up and delivering food shopping and emergency parcels, providing medical supplies and arranging foodbank referrals, offering some digitally mediated company to those feeling lonely while in self-isolation, or simply maintaining corporeal distance to show respect towards the vulnerable. Matthew Bolton, executive director of Citizens UK, noticed that even demographics who perhaps were not as involved in community settings before, recognise the crisis and want to volunteer (in ibid.). More than a testimony of ‘intergenerational solidarity’ during crisis (Lennard 2020), such initiatives also gesture towards what is possible in terms of envisioning and cultivating a kinder, more mindful and more engaged society as a whole, even in the absence of state support and leadership.

A striking example is that of Brazil. The Brazilian government’s response to Covid-19 pandemic has been described as nothing short of catastrophic given its ‘stunning lack of regard for public health […] and the virtual absence of Bolsonaro government’ (Ortega and Orsini 2020: 1258). It was argued that, since the beginning of the coronavirus outbreak, the federal government in Brazil has shown one of the worst responses to the pandemic by failing to develop a national plan for combatting the pandemic and coordinating public health measures, refusing to follow scientific advice and international recommendations and prioritising economic growth over public health concerns (Ferigato et al. 2020; Barberia and Gómez 2020; Lotta et al. 2020). This resulted in Brazil becoming among the three countries with the largest number of confirmed cases and the highest mortality rate (Worldometers 2020), despite efforts by municipal administrations to implement public health interventions at the local level.

Governmental inaction or *laissez-faire*, as Ortega and Orsini (2020: 1263) remind us, is also a form of power that is often underpinned by deliberate efforts to ignore and undermine scientific evidence and a refusal to act on information. This is what McGoey (2012, 2019) refers to as ‘strategic ignorance’ or ‘wilful unknowing’ which has become a form of governance in itself over the last decades and a means of abdicating responsibility and circumventing accountability. So, while some country leaders have been criticised for their excessive interventions and heavy-handed approach to contain the pandemic, the Bolsonaro government, on the other hand, has been criticised for its concerted effort to avoid action; for being ‘“active” in its calculated inaction’ (Ortega and Orsini 2020: 1271).

Interestingly, the lacuna that has been left by the lack of official healthcare leadership in Brazil has been filled, to some extent, by ‘the informal nodes of power that have surfaced in the favelas’ (ibid. 2020: 1259). In Rio de Janeiro, data show that the favelas (slum neighbourhoods) have been one of the epicentres of the disease, figuring a higher coronavirus-related mortality rate among its residents compared to the rest of the city (Garcia 2020). The trend is repeated across other cities in Brazil and exacerbated by the absence of state leadership and assistance, which compelled the mobilisation of numerous independent community groups and favela-based collectives to create a network of support for residents (ibid.). Social projects, such as Corrente do Bem, Semeando Amor Project, Aliança do Bem, Coletivo Semente, NGO Redes da Maré and Jaca Contra o Corona Campaign have been instrumental in gathering and disseminating reliable information about Covid-19 in the community and distributing basic food supplies, personal protective equipment and cleaning materials among the residents of the favelas (Scofano 2020). As reported by Catalytic Communities, a Rio de Janeiro-based NGO and think tank,

since March, hundreds of community groups have implemented widespread communications campaigns using everything from loudspeakers and graffiti, to WhatsApp and podcasts, to inform residents about the virus. They have launched crowdfunding campaigns and drawn on their networks to provide thousands upon thousands of basic food baskets to those hit hardest by the economic shutdown. They have installed public sinks where water access is insufficient and they have been publishing daily on community news portals and social media feeds about the unfolding pandemic in their territories. (CatComm 2020a).

Moreover and in response to extreme underreporting of coronavirus infection and mortality rates in the favelas and the lack of testing, campaigners launched the “Covid-19 in Favelas Unified Dashboard” (www.favela.info) in July 2020 to document the pandemic by collecting data from inside the communities. The dashboard, which is maintained by a network of 20 community collectives, organisations and initiatives, is now the most consistent data source on the spread of the pandemic within Rio de Janeiro’s favelas (CatComm 2020b). It helped raise awareness about the importance of citizen data collection and developing a community response to the underreporting of Covid-19 in Brazil’s most marginalised neighbourhoods. According to Ortega and Orsini (2020: 1269), these local initiatives of mutual aid and grassroot activism ‘defy one-size-fits-all and top-down strategies of strict lockdown and tertiary hospital care adopted in rich countries and offer local alternatives of pandemic control.’ They are not only ‘a hint of what the future awaits but they also can teach the world to tailor measures to tackle epidemics targeted to specific populations […] The community organisation in Rio de Janeiro points to ways to be followed by other vulnerable communities around the world: self-organisation and collective solidarity, not waiting for the state, but seeking to create their own means to overcome adversity’ (Garcia 2020). That said, it is important not to romanticise the role of community initiatives and local civil society in the face of the retreat of the state when it is most needed, as this runs the risk of ‘privatising responsibility’ and putting the onus all the more on vulnerable groups. For although the possibility of an affirmative biopolitics is contingent on solidarity and communal initiatives beyond the state, the state still needs to be positioned as the primary organiser of healthcare and support and pressure must continue to be exerted on governments, holding them accountable for their actions and inactions, especially when their lack of response and concrete action, as witnessed in Brazil, results in mass death and the exacerbation of social inequalities.

## Conclusion

In this paper, I identified and examined two aspects of the political responses to Covid-19 pandemic. The first being the defensive nature of the mechanisms and discourses that have been mobilised in the wake of coronavirus outbreak. From lockdowns and quarantine measures to the deployment of tracking apps and facial recognition systems, the management of the pandemic has been marked by an intensification and acceleration of surveillance techniques and the implementation of emergency powers worldwide. Added to these the adoption of a war rhetoric through which Covid-19 has been continuously likened to an invading enemy which must be defeated through collective effort and social acceptance of various restrictions on everyday freedoms. Drawing on critical literature from the fields of biopolitics and the philosophy of immunology, I argued that such political responses are rooted in the immunitary paradigm of defence in which immunity is understood in terms of the distinction of self (organism) from non-self (what is foreign) and the protection of the inside from the outside. Importantly, such paradigm is not to be understood merely in a biological sense but also as a metaphor for contemporary political governance and social organisation. As Donna Haraway reminds us, ‘the immune system is a map drawn to guide recognition and misrecognition of self and other in the dialectics of western biopolitics. That is, the immune system is a plan for meaningful action to construct and maintain the boundaries for what may count as self and other in the crucial realm of the normal and the pathological’ (Haraway 1991: 204). Seen in this light, the striving for immunity, or what I call “immunitarianism”, inevitably leads to the unleashing of various protective measures, as witnessed during the current pandemic, some of which end up blurring the lines between democracy and totalitarianism, between care and control, between norm and exception, all in the name of defending the population.

Defending the population, nonetheless, is not only a matter of sealing the inside from the outside and erecting protective measures of bordering and securitisation. It also involves exposing some to risk so that the rest can keep on living, a dialectic that is at the heart of modern biopolitics and its power of ‘making live and letting die’, as Foucault argues. One manifestation of this logic can be found in the concept of natural herd immunity through mass infection, which was embraced by some governments (such as Sweden, India and Brazil) as a way of avoiding economic damage that could result out of adopting strict containment measures. Through a critical engagement with this contentious notion of natural herd immunity, the paper addressed the second aspect of the political responses to Covid-19 pandemic, namely their *sacrificial* logic which saw the willingness (both explicit and implicit) of some countries to accept the death of the elderly and the sick by following this type of immunity as an alternative to closing their economies. This sacrificial aspect is also evident in the exposure of those, who do not have the privilege of self-isolation and the option of working from home, to the perils of Covid-19. In the UK, this has led to excess deaths among groups from BAME backgrounds as they are disproportionally represented in frontlines roles and precarious occupations and therefore at a heightened risk of contracting Covid-19. Such reality demonstrates all the more that the (mis-) management of the current pandemic is happening along the same familiar lines of class and racial divisions that are deeply embedded in the rampant exploitative and unequal structures of contemporary capitalist societies. And with the current Covid-19 vaccine rollouts, there are concerns about the possibility of deepening health and economic inequalities even further as a result of supply constraints and the fact that rich countries have already reserved the majority of vaccine doses.

But amidst these defensive and sacrificial aspects lie also affirmative forms of politics and immunity. First and contra the traditional self/non-self paradigm of immunology, treatments such as convalescent plasma therapy demonstrates how self-immunity is not only a matter of defending the self from the intrusion of non-self, but can also depend on the ‘co-presence’ of the other within the self: by conferring the plasma recipient with antibodies derived from someone who has recently recovered from Covid-19, immunity takes on a more cooperative, interconnected and communal dimension that goes beyond the insularity of self/non-self discrimination. Secondly and socio-politically speaking, the various community initiatives that have emerged in response to Covid-19 also demonstrate the possibility of life-affirming solidarity that goes beyond state apparatuses. Brazil is a case in point. Faced with the lack of political leadership and the abandoning inaction of government, numerous social projects and favela-based collectives have been mobilised to provide crucial aid to the vulnerable and disseminate reliable information about Covid-19.

Curiously, what all this seems to highlight is that immunity, with all its defensive and sacrificial logics, does not entirely cancel community altogether but also reaffirms it. This is something that Esposito picks up on, as mentioned before, in his discussions on the radical contradictions inherent within the concept of immunity itself and, by extension, biopolitics as well. As opposed to the orthodox conceptions of immunity as that which is based on the distinction between self/non-self and the negation of community, Esposito contends that immunity and community share ‘a complex dialectic in which neither term is limited to negating the other but instead implicates the other’ (Esposito 2011: 5). Ultimately, he believes that ‘rather than acting as a barrier for selecting and excluding elements from the outside world, [immunity] acts as a sounding board for the presence of the world inside the self’^6^ (ibid.: 169) to the point where immunity becomes ‘indistinguishable from its opposite, “community”: the force of the immune attack is precisely what keeps alive that which it should normally destroy’ (ibid.: 171). While Esposito, here, is explicitly referring to the paradoxical and somewhat enigmatic, case of maternal immune tolerance during pregnancy which enables the inclusion and development of the other (foetus) within the self, the argument can be extended to the wider discussion on community and, more precisely, the inevitability of the mutual and ‘common dependency or vulnerability of one life to another’ (Short 2020), a fact that is amplified all the more during the time of crisis. The Covid-19 pandemic, in this sense, can be seen as an opportunity to rethink the wholesale definitions and practices of identity, community and selfhood beyond the immunitarian principles of self-defence and sacrifice. As Wald (2008: 2) argues, ‘[co]ntagion is more than an epidemiological fact […] The interactions that make us sick also constitute us as a community. Disease emergence dramatizes the dilemma that inspires the most basic of human narratives: the necessity and danger of human contact.’

So, it might be that it is in the midst of crisis that a genuinely affirmative (rather than death-producing) biopolitics could be imagined, lived and shared.

## Footnotes

1 Antigens are proteins that are found on the surface of the pathogen and are unique to that pathogen (Microbiology Society n.d).

2 Antibodies are ‘Y-shaped proteins made by our immune system to protect us against pathogens that have entered the body […] Antibodies are also known as immunoglobulins’ (British Society for immunology 2020). When an antigen enters the body, the immune system produces antibodies against it.

3 See also Agamben (1998) who, contra Foucault, regards biological life as situated within an *originary* relation to politics rather than being imported into it through the advance of modern biopolitics. Life has always been political through and through, according to Agamben.

4 See also Caso’s analysis in ‘Are We at War? The Rhetoric of War in the Coronavirus Pandemic’.

5 Full transcript is available at: https://www.nytimes.com/2020/03/27/podcasts/the-daily/kids-coronavirus.html?showTranscript=1.

6 An argument that also echoes Paradeu’s and Tauber’s remarks on the ecological, interactionist and communal dimension of immunity.
